# Neuropilin-1 and placental growth factor as prognostic factors in metastatic breast cancer

**DOI:** 10.1186/s12885-024-12070-7

**Published:** 2024-03-11

**Authors:** Niina Mäenpää, Leena Tiainen, Mari Hämäläinen, Tiina Luukkaala, Minna Tanner, Outi Lahdenperä, Pia Vihinen, Peeter Karihtala, Pirkko-Liisa Kellokumpu-Lehtinen, Eeva Moilanen, Arja Jukkola

**Affiliations:** 1 Faculty of Medicine and Health Technology, Tampere University, FI-33014 Tampere, Finland; 2 Department of Oncology, Tays Cancer Centre, Tampere University Hospital, FICAN Mid, Teiskontie 35, FI-33521 Tampere, Finland; 3 The Immunopharmacology Research Group, Faculty of Medicine and Health Technology, Tampere University and Tampere University Hospital, Tampere University, 33014 Tampere, P.O. Box 100, Finland; 4 Research, Development and Innovation Center, Tampere University Hospital, Teiskontie 35, FI-33521 Tampere, Finland; 5 Health Sciences, Faculty of Social Sciences, Tampere University, FI-33521 Tampere, P.O. Box 2000, Finland; 6 FICAN West Cancer Centre, Turku University Hospital, 20521 Turku, P.O. Box 52, Finland; 7 Department of Oncology, Helsinki University Hospital Comprehensive Cancer Centre, University of Helsinki, FI-00029 Helsinki, P.O. Box 180, Finland

**Keywords:** Angiogenesis, VEGF, VEGFR, Prognosis, Metastatic breast cancer

## Abstract

**Background:**

Angiogenesis is crucial for tumor development, progression, and metastasizing. The most important regulator of angiogenesis is the vascular endothelial growth factor (VEGF) family, which is involved in multiple pathways in tumor microenvironment. The objective of this study was to investigate the prognostic value of the VEGF family in patients treated for metastatic breast cancer. The emphasis was on neuropilin-1 (NRP-1) and placental growth factor (PlGF).

**Materials and methods:**

An analysis of eight members of the VEGF family was performed using baseline plasma samples of 65 patients treated for metastatic HER2 negative breast cancer in a phase II first-line bevacizumab plus chemotherapy trial. The patients were divided into two groups, high or low, according to the median for each VEGF family member. Progression-free survival (PFS) and overall survival (OS) were determined for each VEGF family member.

**Results:**

The patients with low plasma levels of NRP-1 and PlGF had a longer OS than those with high plasma levels [multivariable adjusted hazard ratios (HRs) 2.54 (95% confidence interval (CI) 1.11–5.82, *p* = 0.02) and 3.11 (95% CI 1.30–7.47, *p* = 0.01), respectively]. The patients with low levels of both NRP-1 and PlGF had a remarkably long OS with HR of 6.24, (95% CI 1.97–19.76, *p* = 0.002). In addition, high baseline NRP-1 level was associated with a significantly shorter PFS [multivariable adjusted HR 2.90 (95% CI 1.02–8.28, *p* = 0.04)] than that in the low-level group, and a high baseline vascular endothelial growth factor receptor-2 level was associated with a longer PFS [multivariable adjusted HR 0.43 (95% CI 0.19–0.98, *p* = 0.04)].

**Conclusion:**

Especially NRP-1 and PlGF have prognostic potential in metastatic breast cancer patients treated with a bevacizumab-taxane combination. Patients with low plasma levels of NRP-1 or PlGF have longer OS than patients with high levels. Patients with both low NRP-1 and PlGF levels appear to have excellent long-term survival.

**Trial registration:**

ClinicalTrials.gov identifier: NCT00979641, registration date 18/09/2009. The regional Ethics Committee: R08142M, registration date 18/11/2008.

## Background

The vascular endothelial growth factor (VEGF) family plays a crucial role in tumor angiogenesis and, thus, in both tumor progression and metastasizing. The VEGF family contains the factors VEGF-A, VEGF-B, VEGF-C, and VEGF-D, placental growth factor (PlGF), and their receptors VEGFR-1, VEGFR-2, VEGFR-3, neuropilin-1 (NRP-1), and neuropilin-2 (NRP-2) [1]. The most important factor for angiogenesis in both normal and malignant tissues is VEGF (also known as VEGF-A), which stimulates angiogenesis through the binding of VEGFR-2 [2].

Neuropilins are multifunctional non-tyrosine kinase receptor proteins. They were first identified as having a crucial role in axonal guidance mediated by binding to semaphorins [3]. Neuropilins and especially NRP-1 bind to VEGF165, an isoform of VEGF-A, and work as co-receptors with VEGFR-2 to facilitate VEGF binding [4]. Neuropilins are expressed on neurons, endothelial cells, epithelial cells, and several types of tumor cells, and their expression is upregulated by hypoxia mediated by VEGF [3, 4]. In addition to binding with VEGF, neuropilins interact with multiple other ligands and pathways in the tumor microenvironment, including interactions with various cells of the immune response [5]. Neuropilins also exist in soluble forms [6, 7]. The soluble form of neuropilin can have either an antagonizing or a promoting role in tumor angiogenesis, depending on its dimerization [5].

PlGF is a glycoprotein that exists in four isoforms (PlGF-1–4), PlGF-1 and PlGF-2 being the predominant isoforms. All isoforms bind to VEGFR-1. PlGF-2 and PlGF-4 also bind to neuropilins [8]. Despite its multiple functions, PlGF does not mediate any critical functions in normal tissue homeostasis, which may be associated with a low expression in many normal tissues. However, it is significantly upregulated in pathologic conditions, such as in many types of cancer, including breast cancer [9, 10]. In addition to cancer cells, many other cell types in the tumor microenvironment produce PlGF, including endothelial cells, fibroblasts, tumor-associated macrophages, and inflammatory cells, and the production is induced by hypoxia and other growth factors. PlGF promotes tumor progression by stimulating angiogenesis and tumor cells directly. By reducing the accumulation of dendritic cells and their functions, PlGF is involved in decreasing antitumor immunologic responses [11].

In this study, we investigated the prognostic value of the plasma concentrations of VEGF family members in patients with metastatic HER2-negative breast cancer who were treated with a first-line taxane-bevacizumab chemotherapy combination.

## Materials and methods

Sixty-five patients with metastatic breast cancer were enrolled into a phase II chemotherapy trial between 2009 and 2013 in three Finnish university hospitals (NCT00979641, registration date 18/09/2009). The study inclusion and exclusion criteria, the trial design, and the primary outcome results have previously been published in detail [12]. Briefly, patients with HER2-negative advanced breast cancer with no previous chemotherapy for advanced disease were treated with a bevacizumab-taxane chemotherapy combination, and the treatment was continued until progression, intolerable toxicity, or patient refusal. After taxane discontinuation, bevacizumab was continued as a maintenance therapy, and for hormone receptor-positive patients, in combination with standard endocrine therapy. After progression, bevacizumab could be continued with a second-line chemotherapy. Informed consent was provided by all patients, and the trial protocol was approved by the Ethics Committee of Tampere University Hospital (R08142M).

### Patient samples and laboratory analysis

Baseline plasma samples were available from 53 of the 65 patients (82%). Baseline plasma samples were collected before starting of the bevacizumab-taxane chemotherapy. The patient characteristics have been published in detail [13]. The characteristics of the patients included in this study are presented in Table 1. Progression-free survival (PFS) and overall survival (OS) were similar at baseline in the population with plasma samples available and in the overall study population.

**Table 1:** Patients’ characteristics and the survival results in the patients with baseline plasma samples available compared with the overall study population

	Baseline plasma population (*n* = 53)	Overall study population (*n* = 65)
Age, years		
Median (range)	58 (32–75)	57 (32–75)
Menopausal status, n (%)		
Pre-menopausal Post-menopausal	8 (15.1) 45 (84.9)	10 (15.4) 55 (84.6)
Hormone receptor status, n (%)		
ER + and/or PR+ ER- and PR-	43 (81.1) 10 (18.9)	53 (81.5) 12 (18.5)
Extent of disease		
< 3 sites ≥ 3 sites	31 (58.5) 22 (41.5)	39 (60.0) 26 (40.0)
Site of metastatic disease, n (%)		
Visceral disease Non-visceral disease	41 (77.4) 12 (22.6)	53 (81.5) 12 (18.5)
Median overall survival, months (95% CI)	39.2 (26.7–51.7)	35.1 (22.2–50.3)
Median progression-free survival, months (95% CI)	11.2 (8.4–14.1)	11.3 (9.7–16.0)

Abbreviations: ER = estrogen receptor, PR = progesterone receptor, CI = confidence interval

Plasma levels of NRP-1, PlGF, VEGF-A, VEGF-C, VEFG-D, VEGFR-1, VEGFR-2, and VEGFR-3 were measured by enzyme-linked immunosorbent assays following the manufacturer’s instructions, using reagents from Invitrogen/eBioscience (Thermo Fisher Scientific, Waltham, MA, USA) for VEGF-A and from R&D Systems Europe (Abingdon, UK) for others. Detection limits were 7.8 pg/ml for neuropilin-1, 1.6 pg/ml for PlGF, 15.6 pg/ml for VEGF-A, 15.6 pg/ml for VEGF-C, 7.8 pg/ml for VEGF-D, 31.3 pg/ml for VEGFR-1, 7.8 pg/ml for VEGFR-2, and 39.1 pg/ml for VEGFR-3.

### Statistical analysis

Based on the baseline levels of NRP-1, PlGF, VEGF-A, VEGF-C, VEFG-D, VEGFR-1, VEGFR-2, and VEGFR-3, patients were divided into the low or high group, using the median value as a cut-off level. Medians and interquartile range (IQR) were reported. Baseline biomarker levels between different baseline characteristics were compared using the Mann-Whitney U test. Cox proportional hazard regression analysis was utilized to calculate hazard ratios (HRs) with 95% confidence intervals (CIs). Multivariable analyses were adjusted for age (continuous), menopausal status, hormone receptor status (positive/negative), the presence of visceral metastases, the number of metastatic lesions at baseline (cut-off three metastatic lesions), and the extent of the disease. Median OS with 95% CI and median PFS with 95% CI were estimated by the Kaplan-Meier method with log rank *p*-values; *p*-values less than 0.05 were considered statistically significant. Statistical analyses were performed using SPSS version 25 (SPSS Inc., Chicago, IL, USA).

## Results

### NRP-1 and PlGF levels and patient baseline characteristics

Baseline NRP-1 levels were similar in groups with different menopausal status (*p* = 0.80), hormone receptor status (*p* = 0.69), and number of metastatic lesions (*p* = 0.80). However, baseline NRP-1 levels were significantly higher in patients with visceral disease or with more than three metastatic sites than in those without visceral disease or with fewer than three metastatic sites [medians 249.9 vs. 192.5 ng/ml (IQRs 185.7–305.4 and 167.9–235.1, *p* = 0.03) and 281.6 vs. 197.3 ng/ml (IQRs 213.7–373.4 and 167.9–257.0, *p* = 0.01), respectively].

Baseline PlGF levels between groups with hormone receptor status (*p* = 0.63), number of metastatic lesions (*p* = 0.06), presence of visceral disease (*p* = 0.68), and number of metastatic sites (*p* = 0.20) were similar. However, the premenopausal patients had significantly lower PlGF levels compared with the postmenopausal patients [medians 19.6 vs. 27.4 pg/ml (IQRs 14.7–20.4 and 21.6–38.1), respectively (*p* = 0.01)].

### Survival results of NRP-1, PlGF, VEGF-A, -C, -D, and VEGFR-1–3

**Fig. 1:**
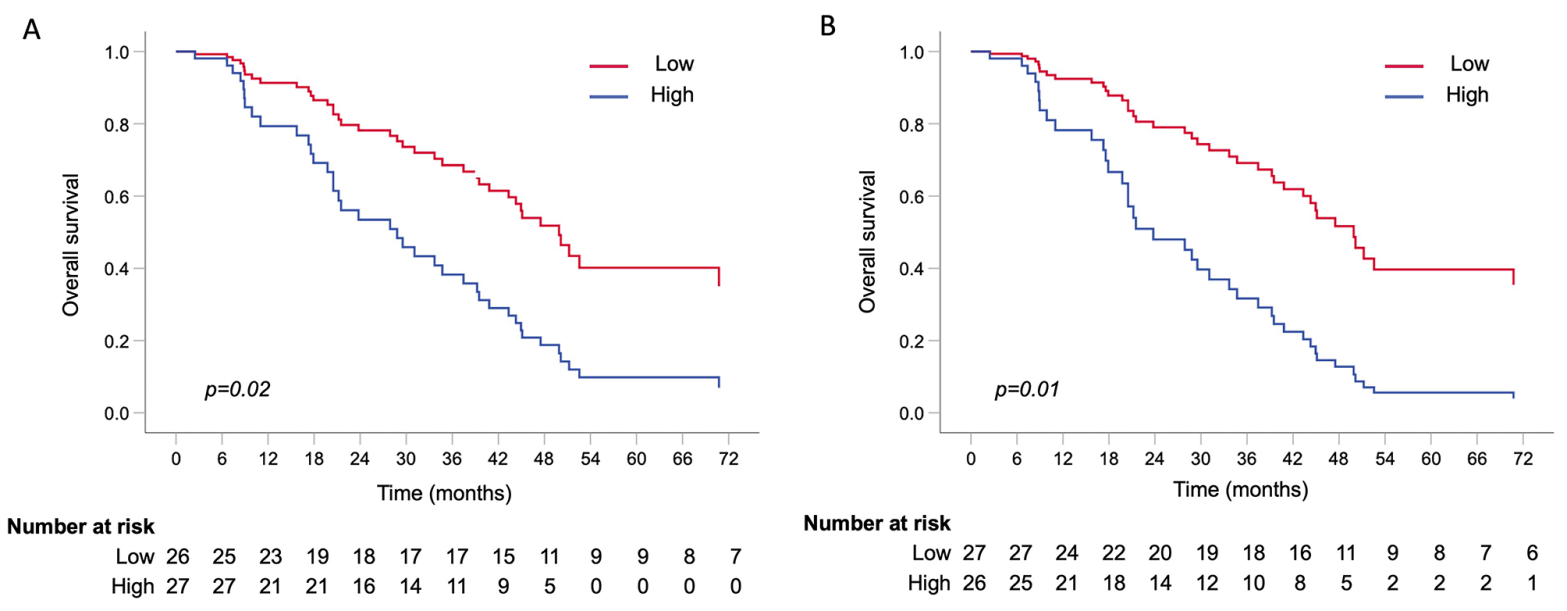
Overall survival in patients grouped by low or high **A**: baseline plasma NRP-1 **B**: baseline plasma PlGF levels using multivariable Cox regression analysis adjusted by age, menopausal status, hormone receptor status, presence of visceral metastasis, number of metastatic lesions and extent of disease

The patients were divided into two groups for each VEGF family member by using the median as the cut-off value (Tables 2 and 3). The patients with low baseline plasma levels of NRP-1 had a significantly longer OS than those with high levels of NRP-1 at baseline [multivariable adjusted HR 2.54 (95% CI 1.11–5.82), *p* = 0.02] (Table 2; Fig. 1A). The median OS for patients with low or high baseline NRP-1 levels was 49.9 months (95% CI NR–NR) and 31.0 months (95% CI 22.8–39.3), respectively, log rank *p* = 0.002. Similarly, the patients with low baseline levels of PlGF had a significantly longer OS than the patients with high levels of PlGF at baseline [multivariable adjusted HR 3.11 (95% CI 1.30–7.47), *p* = 0.01] (Table 2; Fig. 1B). The median OS for patients with low or high baseline PlGF levels was 47.5 months (95% CI 37.1–57.8) and 27.8 months (95% CI 15.9–39.8), log rank *p* = 0.05. In addition, the patients with high baseline VEGFR-1 levels had a worse OS than that of those with low levels when adjusted by age (*p* = 0.05, Table 2). However, in a multivariable model, the survival difference between high and low baseline VEGFR-1-level groups was no longer statistically significant.

**Table 2:** Cox regression analysis for overall survival grouped by low or high baseline levels using the median as a cutoff value

				Overall survival					
		N	n	age-adjusted HR	95% CI	*p* value	multivariable-adjusted HR^a^	95% CI	*p* value
NRP-1	ng/ml								
Low	≤ 236.3	26	13	1			1		
High	> 236.3	27	26	**2.78**	**1.40–5.53**	**0.003**	**2.54**	**1.11–5.82**	**0.02**
PlGF	pg/ml								
Low	≤ 24.7	27	18	1			1		
High	> 24.7	26	21	**2.08**	**0.99–4.37**	**0.05**	**3.11**	**1.30–7.47**	**0.01**
VEGF-A	pg/ml								
Low	≤ 101.9	26	18	1			1		
High	> 101.9	27	21	1.45	0.76–2.75	0.25	1.24	0.62–2.45	0.53
VEGF-C	ng/ml								
Low	≤ 1.6	28	20	1			1		
High	> 1.6	25	19	1.12	0.59–2.11	0.71	1.06	0.53–2.14	0.85
VEGF-D	pg/ml								
Low	≤ 260.5	26	18	1			1		
High	> 260.5	27	21	1.24	0.65–2.39	0.50	0.95	0.45–2.01	0.91
VEGFR-1	pg/ml								
Low	≤ 125.5	27	17	1			1		
High	> 125.5	26	22	**1.90**	**0.98–3.67**	**0.05**	1.10	0.50–2.40	0.79
VEGFR-2	ng/ml								
Low	≤ 11.0	27	20	1			1		
High	> 11.0	26	19	0.77	0.41–1.46	0.43	0.65	0.33–1.28	0.22
VEGFR-3	ng/ml								
Low	≤ 43.5	27	16	1			1		
High	> 43.5	26	23	**2.34**	**1.23–4.47**	**0.01**	1.81	0.88–3.73	0.10

Abbreviations: N = number of patients, n = number of events, HR = hazard ratio, CI = confidence interval

^a^Multivariable-adjusted hazard ratio adjusted by age, menopausal status, hormone receptor status, presence of visceral metastasis, number of metastatic lesions and extent of the disease

**Fig. 2:**
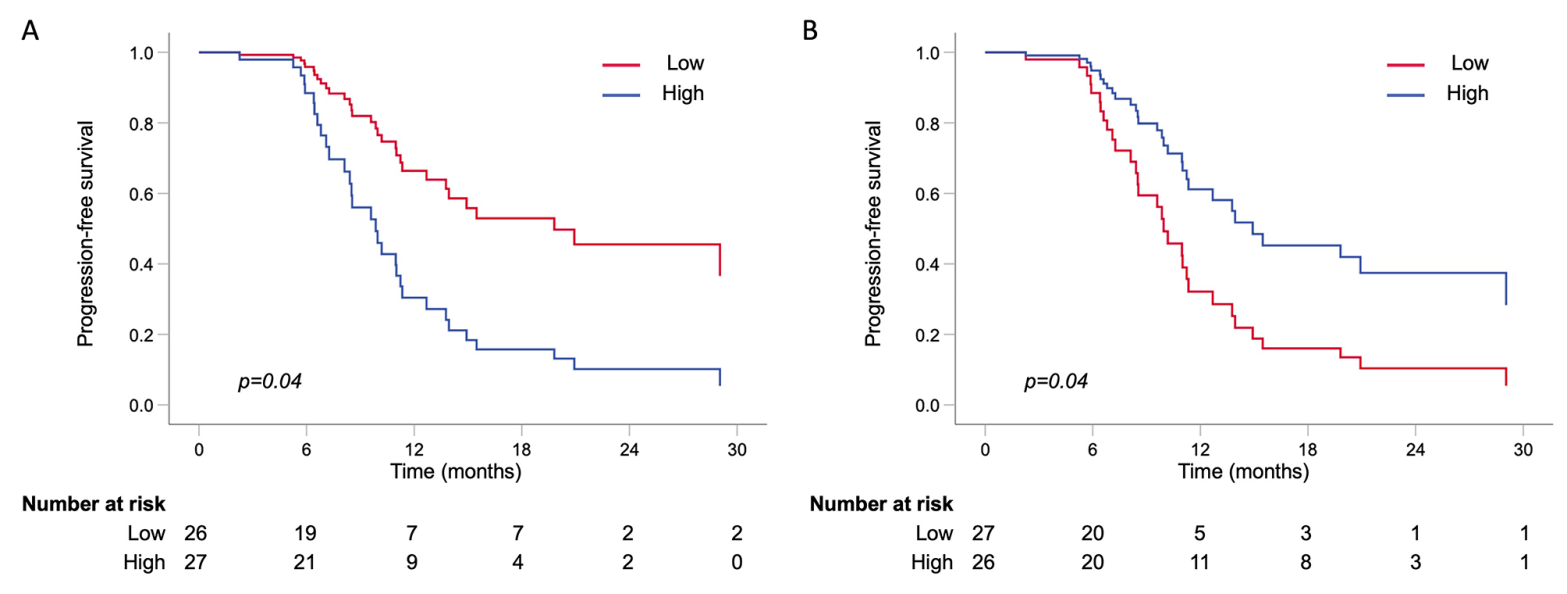
Progression-free survival in patients grouped by low or high **A**: baseline plasma NRP-1 or **B**: baseline plasma VEGFR2 using adjusted multivariable Cox regression analysis adjusted by age, menopausal status, hormone receptor status, presence of visceral metastasis, number of metastatic lesions and extent of disease

High baseline NRP-1 level was associated with significantly shorter PFS compared with low baseline NRP-1 level in the multivariable adjusted analysis [HR 2.90 (95% CI 1.02–8.28), *p* = 0.04] (Table 3; Fig. 2A). The median PFS for patients with a higher NRP-1 level was 11.0 months (95% CI 8.3–13.6) and for patients with a lower NRP-1 was 20.9 months (95% CI 3.9–37.9), log rank *p* = 0.05. However, the patients with high baseline plasma VEGFR2 levels had a significantly longer PFS than those with low VEGFR2 levels by both age-adjusted [HR 0.44 (95% CI 0.21–0.93), *p* = 0.03] and multivariable adjusted analysis [HR 0.43 (95% CI 0.19–0.98), *p* = 0.04] (Table 3; Fig. 2B). The median PFS for patients with high baseline VEGFR-2 levels was 15.4 months (95% CI 6.5–24.4 months) and for patients with low baseline VEGFR-2 levels 9.9 months (95% CI 9.0–10.8 months), log rank *p* = 0.03.

**Table 3:** Cox regression analysis for progression-free survival grouped by low or high baseline levels using the median as a cutoff value

				Progression-free survival					
		N	n	Age-adjusted HR	95% CI	*p* value	Multivariable adjusted HR^a^	95% CI	*p* value
NRP-1	ng/ml								
Low	≤ 236.3	26	10	1			1		
High	> 236.3	27	21	1.98	0.92–4.25	0.07	**2.90**	**1.02–8.28**	**0.04**
PlGF	pg/ml								
Low	≤ 24.7	27	15	1			1		
High	> 24.7	26	16	1.54	0.71–3.32	0.26	1.98	0.84–4.63	0.11
VEGF-A	pg/ml								
Low	≤ 101.9	26	12	1			1		
High	> 101.9	27	19	1.47	0.70–3.10	0.30	1.50	0.69–3.25	0.30
VEGF-C	ng/ml								
Low	≤ 1.6	28	16	1			1		
High	> 1.6	25	15	1.13	0.55–2.32	0.73	1.26	0.58–2.73	0.54
VEGF-D	pg/ml								
Low	≤ 260.5	26	13	1			1		
High	> 260.5	27	18	1.29	0.61–2.70	0.50	0.72	0.30–1.75	0.47
VEGFR-1	pg/ml								
Low	≤ 125.5	27	14	1			1		
High	> 125.5	26	17	1.66	0.81–3.39	0.16	1.56	0.67–3.61	0.29
VEGFR-2	ng/ml								
Low	≤ 11.0	27	19	1			1		
High	> 11.0	26	12	**0.44**	**0.21–0.93**	**0.03**	**0.43**	**0.19–0.98**	**0.04**
VEGFR-3	ng/ml								
Low	≤ 43.5	27	18	1			1		
High	> 43.5	26	13	0.88	0.42–1.82	0.74	0.58	0.23–1.41	0.23

Abbreviations: N = number of patients, n = number of events, HR = hazard ratio, CI = confidence interval

^a^Multivariable adjusted hazard ratio adjusted by age, menopausal status, hormone receptor status, presence of visceral metastasis, number of metastatic lesions and extent of the disease

### Combined analysis of baseline NRP-1 and PlGF levels

**Fig. 3:**
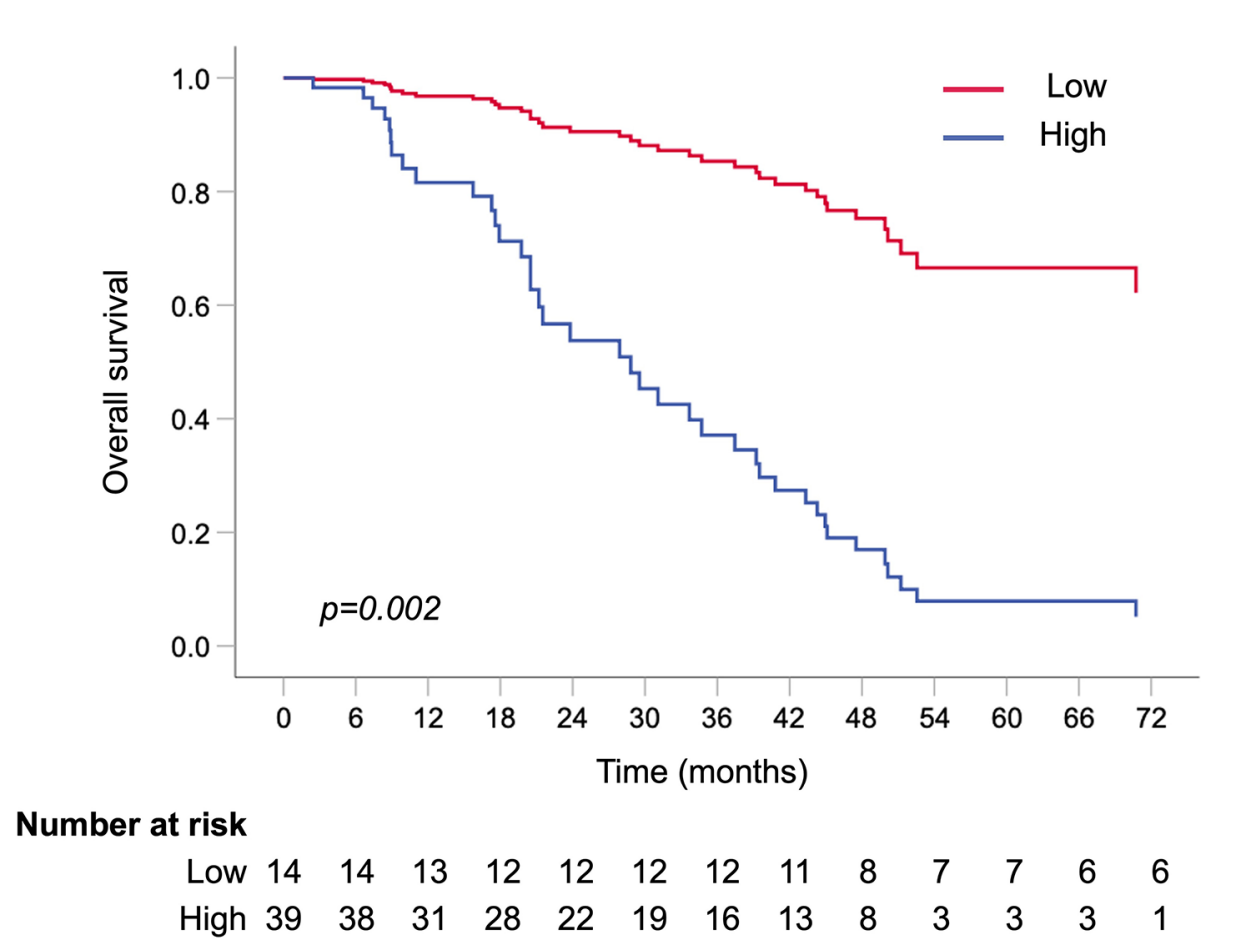
Overall survival in patients grouped by both low NRP-1 and PlGF compared to both or either NRP-1 and PlGF high using multivariable adjusted Cox regression analysis adjusted by age, menopausal status, hormone receptor status, presence of visceral metastasis, number of metastatic lesions and extent of disease

Both low NRP-1 and low PlGF plasma levels were observed in 14 patients (26%), and these patients had a particularly long survival. Using multivariable Cox regression analysis, patients with both low baseline NRP-1 and low baseline PlGF levels had a significantly better OS than the patients with a high level of both or either NRP-1 and PlGF [HR 6.24 (95% CI 1.97–19.76), *p* = 0.002] (Fig. 3). The median OS for patients with both low baseline NRP-1 and low baseline PlGF levels was not reached, and the OS for patients with high either or both NRP-1 or PlGF levels was 29.5 months (95% CI 17.4–41.6), log rank *p* = 0.001.

## Discussion

In the present study, we examined the prognostic value of plasma levels of eight members of the VEGF family in patients with metastatic HER2 negative breast cancer. We discovered that patients with low plasma levels of NRP-1 and PlGF at baseline had significantly longer OS compared to those with high concentrations. The patients with low levels of both NRP-1 and PlGF had an especially long OS. To our knowledge, this study is the first to report the prognostic value of plasma NRP-1 and PlGF levels in advanced breast cancer.

Our results reinforce the prognostic value of high circulating levels of PlGF for poor outcomes in various cancer types previously reported (e.g., in renal cell carcinoma and oral squamous cell carcinoma [14, 15]). Similarly, high tissue expression of PlGF in primarily local breast cancer is associated with poor prognosis (i.e., recurrence, metastasis, and death) in breast cancer [10, 16]. Similar results have also been reported for metastatic cancer. For example, patients with advanced serous ovarian cancer with high tumor tissue expression of PlGF have shorter OS than patients with low tumor tissue PlGF expression [17]. The association between higher levels of PlGF and poorer outcomes in malignancies is probably related to the fundamental role of PlGF in tumor angiogenesis and immunosuppression. In our study, there was a correlation between high levels of PlGF and shorter OS, but a similar effect was not seen in PFS. There was no significant difference in PFS between patients with high or low PlGF levels. The reason there was no effect on PFS with different levels of PlGF is unclear.

The prognostic role of circulating NRP-1 levels has not previously been studied in metastatic breast cancer. Poorer outcomes related to high NRP-1 levels have been reported for several tumor types and also in early breast cancer [18–22]. Of note, NRP-1 has been studied as a prognostic factor mainly in early breast cancer by using immunochemistry. However, circulating NRP-1 levels were studied in early breast cancer, and patients with low NRP-1 levels at baseline had a significantly better breast cancer-specific survival than patients with high NRP-1 [19]. Also, the levels of both circulating NRP-1 and tumor tissue expression of NRP-1 increase in advanced nodal and metastatic breast cancer compared to local disease [20].

In addition to tumor development through angiogenesis, poorer outcomes with higher circulating NRP levels might be related to more drug-resistant tumors. NRP-1-mediated upregulation of other growth factor pathways can be a mechanism for acquired resistance (e.g., in HER2-targeted therapies [23]). In murine non-small cell lung carcinoma models, targeting NRP-1 with anti-NRP simultaneously with anti-VEGF therapy resulted in more effective tumor growth reduction [24].

We can only hypothesize if low PlGF or NRP-1 levels are also associated with bevacizumab efficacy. All the patients in our study were treated with anti-VEGF-antibody bevacizumab combined with chemotherapy. To date, no clear indicators (e.g., biomarkers) have been established to select the patients most likely to benefit from bevacizumab, neither in breast cancer nor in other cancer types [25]. Because our study did not have a control arm, the effect of bevacizumab on survival remains unclear. In addition to the lack of a control arm, another limitation of our study was the overall small number of subjects participating in the study. Because of the limitations, further randomized, prospective studies are needed on the topic.

As was stated, many other studies have used tumor tissue samples in their analyses. In our study, we analyzed plasma samples to evaluate the concentrations of the VEGF family members. In metastatic breast cancer, tumor tissue collection can be challenging, but obtaining plasma samples is a feasible and non-invasive method.

Additionally, we explored other angiogenetic factors. Of these, VEGFR-2 was the only one with statistically significant PFS difference between patient groups. The patients with a high baseline plasma VEGFR-2 concentration had significantly longer PFS than those with a low baseline level. Similar results were presented in an earlier study examining bevacizumab efficacy in metastatic breast cancer [26]. High baseline VEGFR-2 levels are also associated with bevacizumab efficacy as adjuvant therapy [27].

## Conclusion

Our findings suggest that plasma NRP-1 and PlGF are useful in evaluating the prognosis of patients with advanced HER2-negative breast cancer receiving bevacizumab and taxane. Further prospective studies are warranted on the topic in a larger patient population. Since angiogenesis and its complex pathways are crucial for tumor development and metastasis, both NRP-1 and PlGF may be attractive treatment targets in the future.

## Data Availability

The datasets used and analyzed during the current study are available from the corresponding author on reasonable request.

## Abbreviations

NRP: Neuropilin
PlGF: Placental growth factor
sNRP: Soluble neuropilin
VEGF: Vascular endothelial growth factor
VEGFR: Vascular endothelial growth factor receptor
